# CD95 ligand induces senescence in mismatch repair-deficient human colon cancer via chronic caspase-mediated induction of DNA damage

**DOI:** 10.1038/cddis.2017.87

**Published:** 2017-03-16

**Authors:** Danielle A Raats, Nicola Frenkel, Susanne J van Schelven, Inne HMBorel Rinkes, Jamila Laoukili, Onno Kranenburg

**Affiliations:** 1 Cancer Center, University Medical Center Utrecht, Heidelberglaan 100, Utrecht 3584CX, The Netherlands

## Abstract

CD95 is best known for its ability to induce apoptosis via a well-characterized pathway involving caspase-mediated proteolytic events. However, in apoptosis-resistant cell lines of diverse cancer types stimulation of CD95 primarily has pro-tumorigenic effects that affect many of the hallmarks of cancer. For instance, in colon cancer cells with a mutant *KRAS* gene CD95 primarily promotes invasion and metastasis. In the current study, we further investigated the context dependency of the consequences of CD95 activation in colon cancer. We used a series of patient-derived three-dimensional colon cancer cultures and studied their response to stimulation with CD95 ligand (CD95L). CD95L had a strong inhibitory effect on the clone-forming capacity of five out of nine cultures. In line with previous work, these cultures all had a wild-type *KRAS* gene and expressed high levels of CD95. Furthermore, the most sensitive cultures were characterized by microsatellite instability (MSI) and deficient mismatch repair. The reduced clonogenic growth of MSI-type colonospheres resulting from chronic CD95 stimulation was only partly due to apoptosis as many tumor cells survived treatment, yet were unable to regenerate clones. CD95 stimulation caused an irreversible cell cycle arrest, which was associated with cytokine secretion, similar to the senescence-associated secretory phenotype (SASP), and expression of senescence-associated *β*-galactosidase. In human colon cancer cohorts, CD95 expression was strongly correlated with the recently identified consensus molecular subtype 1 (CMS1), which mainly consists of MSI-high tumors, and with two independent SASP signatures. Mechanistically, CD95-induced senescence was caused by chronic DNA damage via caspase-activated DNAse resulting in p53 activation and p21 expression, with a minor contribution of the SASP. We conclude that induction of senescence is a hitherto unrecognized consequence of high CD95 expression, which appears to be most relevant for CMS1.

CD95 is a cell surface receptor with pleiotropic context-dependent functions. For instance, it is essential for removing self-reactive T cells or activated peripheral T cells during the termination phase of an immune response.^1, 2^ The role of CD95 in T-cell homeostasis is largely ascribed to its capacity to activate the classical caspase cascade, which leads to programmed cell death.^1^ However, CD95 is widely expressed outside the T-cell compartment and regulates multiple physiological processes through the activation of non-apoptotic signaling pathways.^3^ These include the stimulation of hepatocyte proliferation during liver regeneration,^4^ the activation of neural stem cells during memory formation and brain repair,^5^ and neutrophil recruitment during inflammation.^6^ Upon binding of CD95 to its ligand – CD95L – several non-caspase signaling pathways can be activated, resulting for instance in the activation of Src family kinases and receptor tyrosine kinases.^6, 7, 8, 9, 10^

If the prime consequence of CD95 activation is to induce apoptosis, one would expect tumor cells to silence the pathway. Indeed, caspase-8 mutations are found in a small subset (~4%) of colon tumors, disabling extrinsic apoptosis induction.^11, 12^ However, the expression of CD95 itself is retained in the vast majority of colon tumors (this report). In many cases, the expression of CD95L is even increased with tumor progression, suggesting that non-apoptotic signaling is dominant in the context of (colon) cancer development.^3^ CD95 stimulation can promote tumorigenesis by stimulating tumor cell invasion,^8, 9, 13, 14, 15^ proliferation,^16^ epithelial–mesenchymal–transition (EMT),^17^ and cancer stem cell maintenance.^18^ Recent data further suggest that there is a general requirement for CD95 and CD95L expression for tumor cell survival across tumor types,^19^ although the key signaling pathways underlying this dependency are currently not known. As CD95 has pro-tumorigenic effects in many apoptosis-resistant cancer types – including glioblastoma and colon cancer – the concept of CD95 inhibition as an anti-cancer strategy has emerged.

Aside from a general requirement for CD95 and CD95L for tumor cell survival, most of the tumor-promoting activities of CD95 are likely to be context dependent. For instance, we have previously shown that the presence of oncogenic KRAS in colon cancer cells causes a switch in CD95-signaling output from apoptosis to invasion, which is required for metastatic spread and tumor recurrence.^15, 20, 21^ This raises the question of how to select patients for CD95-inhibitory therapy and whether there could be genetic or epigenetic traits that may determine the response to such therapy. To address this, we further explored the context dependency of CD95 signaling in human colon cancer by making use of a series of patient-derived ‘colonosphere' cultures.

We describe a novel tumor-suppressive function for CD95 involving chronic activation of the caspase cascade at sub-apoptotic levels, leading to sustained DNA damage and the induction of p53-dependent senescence. This was largely restricted to *KRAS* wild-type cultures and was strongly associated with deficient mismatch repair (dMMR) in human colon cancer. The results imply that the development of CD95-targeted therapy for the treatment of human colon cancer holds the potential danger of interfering with an intrinsic tumor suppressor mechanism in a specific subset of human colon tumors.

## Results

### CD95 ligand induces an irreversible cell cycle arrest in colon cancer cells with high expression of CD95

**Figure 1:**
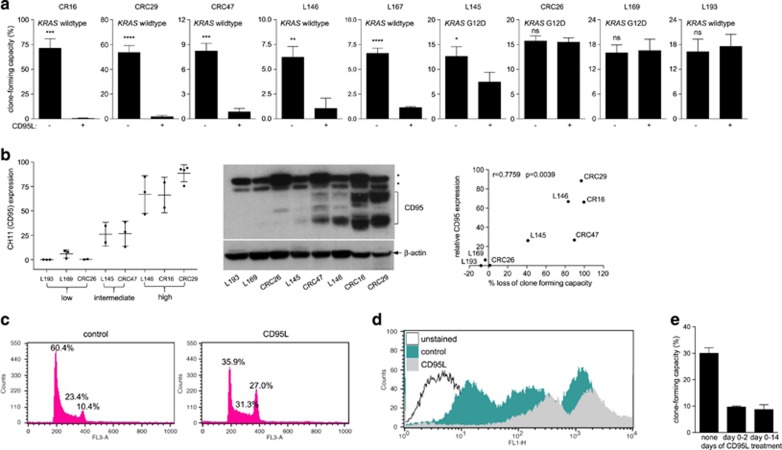
Chronic CD95 stimulation reduces colony-forming capacity in human colonospheres. (**a**) Human colonosphere cultures isolated from the tumors of nine distinct colon cancer patients were pretreated with FC control or FC-CD95L (10 ng/ml) for 2 weeks, seeded as single cells in Matrigel and cultured continuously in the absence or presence of FC-CD95L. Colony formation was scored after 2 weeks. The experiments were performed multiple times with similar results (L145: *n*=4, L146: *n*=4, L167: *n*=3, CRC47 *n*=4, CRC29 *n*=3, L193 *n*=2, CRC26 *n*=1, L169 *n*=4, CR16 *n*=3). One representative experiment is shown (triplicate values). (**b**) The expression of CD95 was measured with FACS (CH11, left panel) and with western blotting (middle panel; asterisks indicate non-specific bands). The FACS results are plotted as the percentage CH11-positive cells over control for each cell line. Averaged FACS values were then plotted against the percentage growth inhibition (right panel). The Pearson correlation and the accompanying *P*-value are shown in the inset. (**c**) CRC29 cells were cultured in the presence of FC control or FC-CD95L for 2 weeks and were then processed for FACS analysis of DNA content using PI. (**d**) CRC29 cells were cultured in the presence of control or CD95L for 2 weeks, labeled with CFSE, and subsequently plated in Matrigel as single cells. Cells were collected from the Matrigel and analyzed for maintenance of the fluorescent signal by FACS at days 2, 4, and 7 after plating. (**e**) The experiment was performed as in **a**, comparing a 2-day treatment to chronic stimulation with FC-CD95L during colony formation

We have previously shown that CD95L stimulates migration, invasion, and metastasis of KRAS-mutant apoptosis-resistant colorectal tumor cell lines.^15^ To assess the context dependency of these findings, we determined the effect of chronic CD95 activation on a series of nine patient-derived colonosphere 3D cultures. First, we analyzed whether a 2-week stimulation with CD95L had an effect on the clonogenic capacity of such cultures. We found that chronic CD95L exposure suppressed the colony-forming potential of five out of nine of these cultures by more than 50% (Figure 1a). We found a strong correlation between the level of CD95 protein expression by FACS and western blotting and the ability of CD95L to suppress clone-forming capacity (Figure 1b and Supplementary Figure S1).

Strikingly, the reduction in clone-forming capacity could not be explained by apoptosis as CD95L stimulation had no effect on the ratio of live : dead single cells seeded in Matrigel as quantified by calcein staining and ethidium bromide uptake (Supplementary Figure S2). However, FACS analysis of PI-stained cells did show a marked increase in the number of cells in G2 (Figure 1c). Moreover, pulse-chase FACS analysis of CFSE-labeled cells showed that CD95L stimulation inhibited the expansion of living single cells in Matrigel (Figure 1d).

Next, we assessed whether the cell cycle arrest induced by CD95L, and the resulting loss of clonogenic capacity, was reversible. To this end, tumor cells in Matrigel were exposed to CD95L for 2 days after which it was washed away and colony formation was allowed to occur for two subsequent weeks. Figure 1e shows that a 2-day incubation with CD95L was sufficient to induce an irreversible cell cycle arrest and prevent colony outgrowth.

To exclude the possibility that the observed effects were non-specific, we used a soluble form of the ligand-binding domain of CD95 (CD95-Fc), which neutralizes CD95L. In addition, we generated CD95 knockdown cells. We found that CD95L neutralization (Supplementary Figure S3A) or CD95 knockdown (Supplementary Figures S3B and S3C) both prevented CD95L-induced cell cycle arrest.

**Figure 2:**
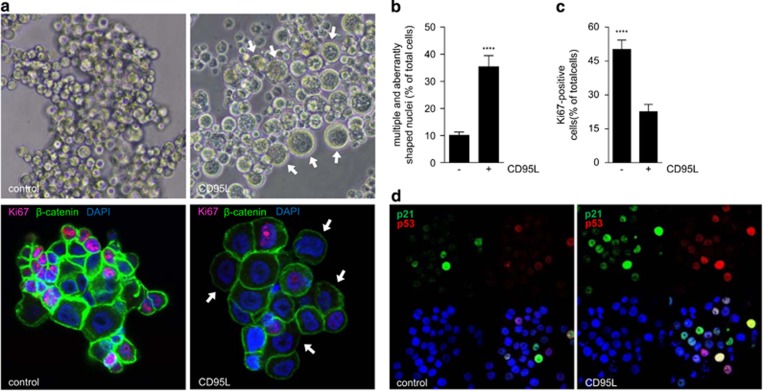
CD95 stimulation causes reduced proliferation and induction of p53. (**a**) Bright field images of CR16 cells (upper panel) show the increase in cell size (indicated by white arrows) following 1-week treatment with FC or FC-CD95L. Immunofluorescence analysis (lower panel) for Ki67 (red), *β*-catenin (green) and nuclei (DAPI, blue) of control and CD95L-treated CRC29 colonospheres. Cells were chronically treated for 2 weeks, plated as single cells in Matrigel in the absence or presence of CD95L for 7 days. (**b**) Quantification of the number of cells with aberrantly shaped or multiple nuclei in control and CD95L-treated CRC29 cultures treated as in (**a**). (**c**) Quantification of the number of Ki67-positive cells in control and CD95L-stimulated CRC29 colonospheres treated as in (**a**). (**d**) Immunofluorescence analysis of p53 and p21 induction in CRC29 colonospheres treated for 24 h with FC control and FC-CD95L. ***P<0.00001

We next analyzed the colonies that did form in the presence of CD95L. Immunofluorescence analysis showed that CD95L treatment caused an increase in cell size (Figure 2a), an increase in the percentage of cells with multiple and/or aberrantly shaped nuclei (Figures 2a and b), a decrease in the number of Ki67-positive cells (Figures 2a and c) and an increase in the number of p53- and p21-positive cells (Figure 2d). These results demonstrate that CD95L induces a powerful anti-proliferative signal in the subset of colorectal spheroid cultures with high expression of CD95.

### CD95 ligand induces senescence through chronic caspase-dependent induction of DNA damage and activation of p53

**Figure 3:**
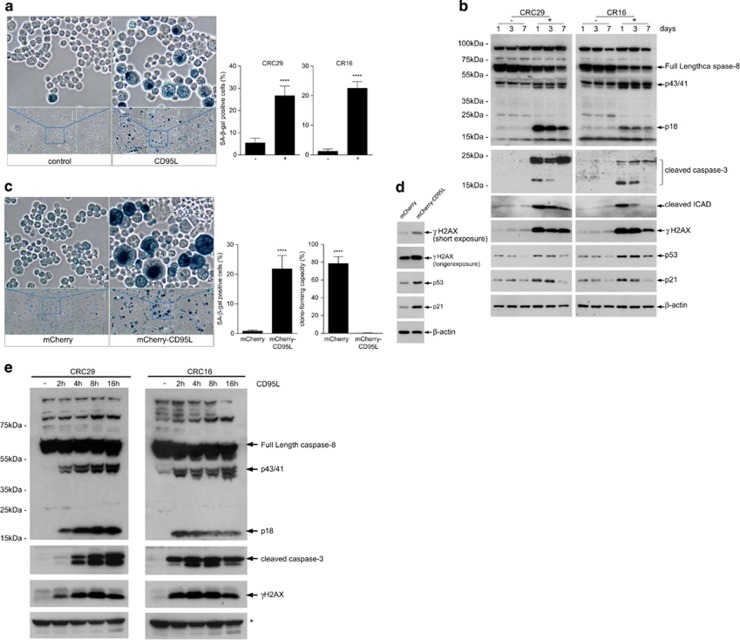
CD95L induces senescence in human colonospheres. (**a**). Human colonospheres (CRC29, CR16) were either treated with FC control or with 10 ng/ml of FC-CD95L for 7 days. Control and CD95L-treated cells were then analyzed for SA-*β*GAL activity. Significance (unpaired *t*-test): *P*<0.0001; CR16 *P*<0.0001. (**b**) Human colonospheres (CRC29, CR16) were treated with FC control (−) or 5ng/ml FC-CD95L (+) for 1, 3, or 7 days. Western blot analysis of caspase-8 and -3 activation, cleavage of iCAD, and the DNA damage marker γH2AX over time. (**c**) A lentiviral vector for expression of mCherry-CD95L or a control vector (mCherry backbone without CD95L) was introduced into human CRC29 colonospheres. Ten days after transduction, SA-*β*GAL activity was measured as in **a**. Significance (unpaired *t*-test): *β*GAL assay *P*<0.0001; colony-forming assay: *P*<0.0001. (**d**) CRC29 cells expressing either mCherry or mCherry-CD95L (as in **c**) were analyzed by western blotting for the presence of γH2AX, p53, and p21 induction. (**e**) Human colonospheres (CRC29, CR16) were either treated with FC control (−) or were with 10 ng/ml of FC-CD95L for the indicated time points. Western blot analysis was then used to assess caspase-8 and -3 activation and the DNA damage marker γH2AX over time. * indicates unspecific band

The irreversible nature of the growth arrest (Figure 1e) suggested to us that CD95L may induce senescence. Indeed, we found that CD95L stimulation induced expression of senescence-associated *β*-galactosidase (SA-*β*GAL) (Figure 3a). CD95L also caused chronic elevation of γH2AX, a marker of DNA damage and senescent cells,^22^ and stabilization of p53 (Figure 3b). Endogenous production of CD95L from an expression construct also severely reduced clonogenic capacity and induced the expression of SA-*β*GAL (Figure 3c). This was accompanied by induction of the DNA damage marker γH2AX, stabilization of p53, and expression of its target p21 (Figure 3d). A time course experiment showed that induction of γH2AX following CD95L stimulation paralleled caspase-8 and caspase-3 activation, starting already 2 h following ligand stimulation (Figure 3e).

Next, we analyzed the signaling requirements for CD95L-indcued senescence and reduction in clone formation. One of the effectors that is activated downstream of CD95 is the ROS-generating enzyme NOX.^23^ As ROS production can contribute to cellular senescence, we considered the possibility that CD95-induced senescence was due to ROS production. However, treatment with the ROS scavenger *N*-acetylcysteine increased the general clone-forming capacity (as we have shown before^24^) but had no effect on CD95L-induced loss of clonogenic capacity (Supplementary Figure S4), indicating that ROS production is dispensable for CD95L-induced senescence.

**Figure 4:**
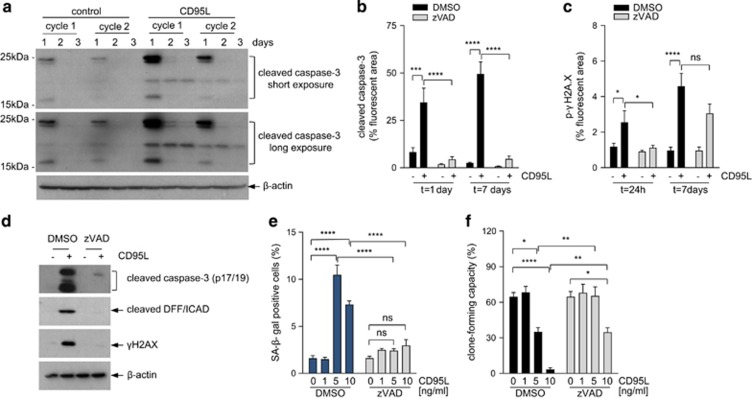
CD95-induced senescence is mediated by low-level canonical caspase signaling. (**a**) CRC29 colonospheres were exposed once (cycle 1) or chronically exposed to CD95L (10 ng/ml) for 2 weeks (cycle 2), and collected at the indicated time points. Cells were collected and analyzed for the presence of activated (cleaved) caspase-3 by western blotting. (**b**–**c**) CRC29 colonospheres were exposed to CD95L (5 ng/ml) in the presence or absence of zVAD (25 *μ*M) for 1 or 7 days and were then processed for imunofluorescence analysis of caspase-3 activation (**b**) and DNA damage (γ-H2AX) (**c**) An overview of the significance of all comparisons is provided in Supplementary Table S1. (**d**) Colonspheres were exposed to CD95L (10 ng/ml) for 24 h in the presence or absence of zVAD (25 *μ*M) followed by western blot analysis of caspase cleavage and iCAD processing. (**e** and **f**) CRC29 colonospheres were exposed to increasing concentrations of CD95L as indicated in the presence or absence of zVAD (25 *μ*M), after which SA-*β*GAL (**e**) and clone-forming capacity (**f**) were assessed at 7 or 14 days, respectively. The asterisks indicate significant differences (ordinary one-way ANOVA) (****P<0.00001; **P<0.001). An overview of the significance of all comparisons is provided in Supplementary Table S1

We observed that long-term CD95L stimulation was associated with caspase cleavage at a much lower level than during the immediate response (Figure 4a). Caspases induce DNA damage by cleaving the inhibitor of caspase-activated DNAse (iCAD)^25^ and this could be an alternative mechanism of ligand-induced DNA damage. Indeed, we found iCAD processing and induction of the DNA damage marker γH2AX during CD95L-induced senescence (Figures 3b and 4c). To test whether CD95L induces senescence through a mechanism involving caspase/CAD-mediated DNA damage, we used the caspase inhibitor zVAD. zVAD completely blocked CD95L-induced caspase-3 activation (Figures 4b and d) and also prevented CD95L-induced iCAD cleavage (Figure 4d), DNA damage (Figures 4c and d), and induction of SA-*β*GAL (Figure 4e). As a result, zVAD nearly completely restored colony-forming potential in the presence of CD95L (Figure 4f). To further test caspase involvement in CD95-induced senescence, we performed rescue experiments in CD95 knockdown cells using either wild type (full length) or death domain-deleted (ΔDD) CD95, which is incapable of caspase activation. As expected, CD95 knockdown protected cancer cell sensitivity to CD95L-induced loss of clone-forming capacity (Supplementary Figures S5A and S5B). Expression of full-length shRNA-insensitive CD95 in CD95 knockdown cells restored sensitivity to CD95L, but expression of ΔDD–CD95 failed to do so (Supplementary Figures S5A and S5B). Strikingly, expression of ΔDD–CD95 also protected CD95L-stimulated control cells against CD95L, thus interfering with signal transduction by endogenous CD95. Indeed, ΔDD–CD95 completely prevented CD95L-induced caspase activation in control (CD95-expressing) cells (Supplementary Figures S5A and S5B).

**Figure 5:**
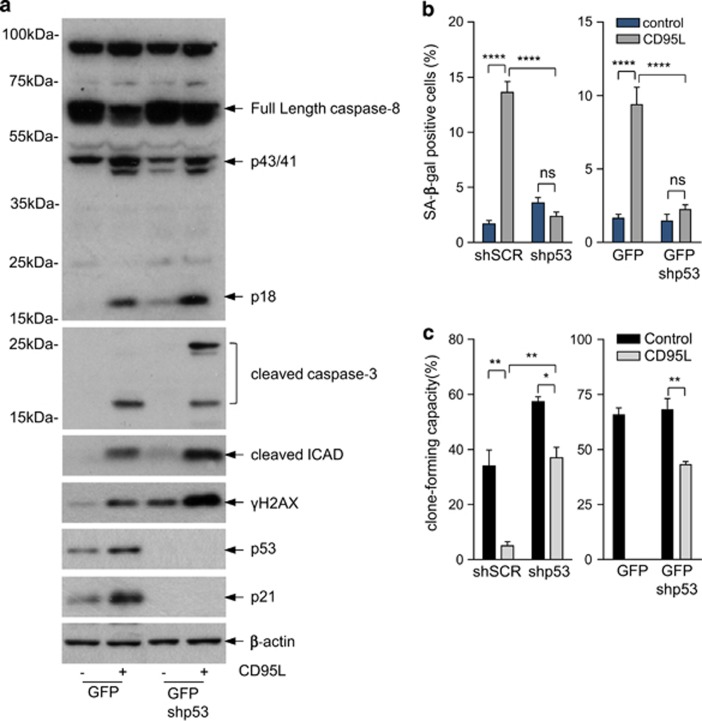
CD95-indcued senescence depends on p53. (**a**) CRC29 colonospheres were transduced with a lentiviral vector expressing control shRNA or shRNA-targeting p53, and were subsequently exposed to FC or FC-CD95L. CD95L-induced activation of caspase-8, caspase-3, cleaved iCAD, DNA damage (γ-H2AX), and p21 induction were then assessed by western blotting. (**b** and **c**) Control CRC29 and two independent clones of p53-suppressed colonospheres were exposed to FC-CD95L for 14 days, and analyzed for SA-*β*GAL activity (**b**) and for clone-forming capacity (**c**). The asterisks indicate significant differences (ordinary one-way ANOVA) (****P<0.00001; **P<0.001; *P<0.01). An overview of the significance of all comparisons is provided in Supplementary Table S1

To assess the role of p53 in CD95L-induced senescence, we generated p53 knockdown cells. Knockdown of p53 did not prevent upstream events like caspase-8 and caspase-3 activation, iCAD processing or DNA damage induction. Rather, p53 knockdown increased CD95L-induced DNA damage (Figure 5a), which is in line with its function as a ‘guardian of the genome' by activating genes involved in cell cycle arrest and DNA repair.^26^ Despite the increased DNA damage in CD95L-stimulated p53 knockdown cells, these cells did not enter senescence (Figure 5b) and largely retained their clone-forming potential (Figure 5c).

Together, these results identify senescence as a novel CD95-signaling output in tumor cells through chronic activation of caspase-induced DNA damage.

### Mutant KRAS protects tumor cells against CD95-induced senescence

We have previously shown that the presence of oncogenic mutations in the *KRAS* gene interferes with caspase-mediated apoptosis induction by CD95.^15^ In line with this, we found that all five CD95L-sensitive colonosphere cultures had a wild-type *KRAS* gene (Figure 1a and Supplementary Table S2). We next stimulated isogenic cells differing only in the presence/absence of an endogenous *KRAS* oncogene with CD95L and measured colony-forming potential, apoptosis, and senescence. Stimulation of a murine *KRAS*-mutant colorectal tumor cell line (C26) with CD95 ligand caused an increase in colony-forming potential (Supplementary Figure S6A). By contrast, chronic stimulation of *KRAS*-deficient C26 cells (L13 cells) caused a pronounced growth inhibition, which was associated with the induction of SA-*β*GAL (Supplementary Figures S6B and S6C). We conclude that mutant *KRAS* not only protects tumor cells against CD95L-induced apoptosis, but also against CD95L-induced senescence. This in line with the notion that both phenomena require activation of the caspase cascade and that this is suppressed in the presence of mutant KRAS.^15^

### CD95L induces a secretory phenotype which contributes to reduced clone formation

**Figure 6:**
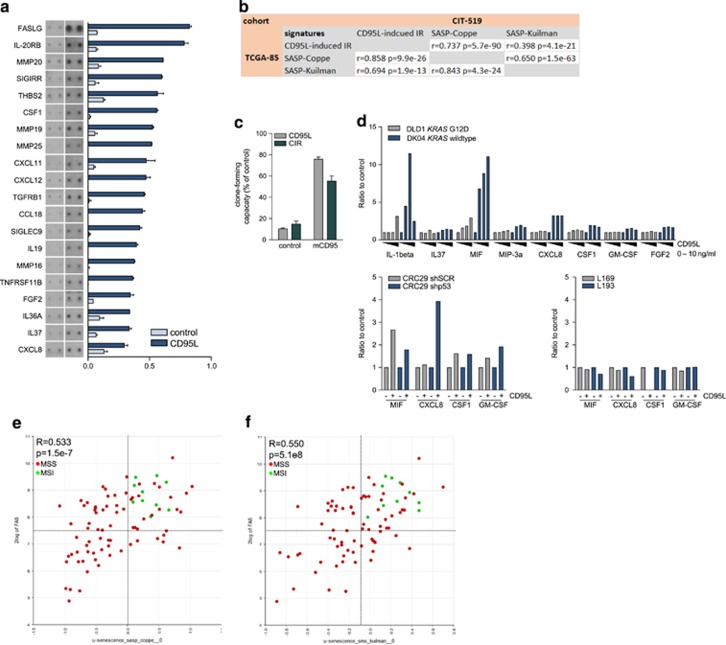
CD95L induces a SASP, which correlates with MSI in colon cancer. (**a**) Analysis of the conditioned medium of control- and CD95L-stimulated CRC29 colonospheres using cytokine array. (**b**) Expression of the corresponding CIR gene set was then correlated with expression of the two SASP gene sets in two distinct cohorts (TCGA-85 (COAD 2000-01-01) and CIT-519 (gse39582)). (**c**) The contribution of the CIR to CD95L-indcued suppression of clonogenic capacity was tested by using the extracellular part of murine CD95 (mCD95) as a trap for the added ligand. Conditioned medium was collected from control or FC-CD95L-treated CRC29 clones and incubated with or without mCD95. Untreated CRC29 cells were then plated as single cells in Matrigel and treated for 2 weeks with control or mCD95-treated conditioned medium, and subsequently analyzed for their clone-forming capacity. (**d**) DLD1 and DKO4 cells (upper graph) were treated with FC control or increasing concentrations of FC-CD95L (2, 5, or 10 ng/ml). Conditioned medium was collected 2 or 7 days after treatment and analyzed by Luminex. CRC29 colonospheres expressing control shRNA or shp53 (left lower graph), L169 and L193 colonospheres (right lower graph) were pretreated with FC or FC-CD95L (10 ng/ml) for 7 days, and plated as single cells in Matrigel. Conditioned medium was collected 2 or 4 days after treatment and analyzed by Luminex. (**e** and **f**) The *xy* plots show the correlation between expression of CD95 and two independent SASP signatures^28, 29^ in a data set with annotated MSI status (TCGA, COAD; 2000-01-01)

Previous work has shown that a senescence-associated secretory phenotype (SASP) is instrumental in inducing and maintaining the senescent phenotype.^27, 28, 29^ Therefore, we tested whether CD95L would induce a SASP-related secretory phenotype. Human colonospheres were stimulated with CD95L and the conditioned medium was analyzed by cytokine arrays. We found that CD95L stimulation induced the secretion of a set of 19 cytokines, including M-CSF, SDF1/CXCL12, and IL8, which we termed the CD95L-induced inflammatory response (CIR) (Figure 6a and Supplementary Table S3). Expression of the CIR in human colon cancer cohorts was strongly correlated with expression of two independent SASP signatures^28, 29^ (Figure 6b and Supplementary Table S3). In addition, expression of CD95 itself was also significantly correlated with both SASP signatures (Table 1). We next tested whether CIR-containing medium was able to reduce clonogenic capacity in the absence of CD95L. To this end, medium was collected from CD95L-induced senescent cells. The CIR-containing medium significantly reduced the clonogenic capacity of colonospheres. Blocking residual CD95L in the CIR medium restored clone-forming efficiency to ~60% of control (Figure 6c). These data show that the CIR contributes to CD95L-induced reduction of clonogenic capacity, but also that the direct action of CD95L on CD95 is dominant.

Next, we tested whether the CIR was dependent on p53 or KRAS status. To test this, we used isogenic cell pairs differing only in the presence of mutant KRAS (DLD1 *versus* DKO4) or p53 (CRC29 +/− shp53). In addition, as negative controls we used cell lines L169 and L193, which do not express CD95 and did not show a CIR (Figure 1). Luminex analysis of senescence-associated cytokines in the conditioned media showed that CIR was completely suppressed in the presence of mutant KRAS (Figure 6d). However, in the absence of p53 it was maintained (Figure 6d), despite the fact that such cells fail to senesce in response to CD95L. This suggests that mutant KRAS blocks both CD95-induced senescence and SASP/CIR, but that p53 regulates senescence but not the SASP/CIR. The experiments with the p53 knockdown cells also show that the SASP/CIR contributes but is insufficient to induce senescence in the absence of p53. This is in line with the results in Figure 6c, showing only a minor contribution of the SASP/CIR to CD95L-induced senescence.

### CD95L-induced senescence correlates with microsatellite instability and CMS1

We noted that two of the colonosphere lines that were most sensitive to CD95L-indcued senescence display microsatellite instability (MSI) (Supplementary Table S2). Colon tumors with MSI constitute a distinct molecular entity. Gene expression profiling has shown that tumors with MSI are highly enriched in the consensus molecular subtype 1 (CMS1).^30^ To assess a potential relationship of CD95 expression with MSI status, CMS1, or other colon cancer subtypes in an unbiased fashion we analyzed CD95 expression in a large cohort for which the consensus classification was available (gse39582).^30^ In addition, we generated a list of 200 genes that were most significantly co-expressed with CD95 (the CD95 ‘neighborhood') as an alternative tool to analyze the correlation of CD95 expression with other clinical and genetic parameters (Supplementary Table S4). First, we analyzed the correlation of CD95 expression with a series of gene signatures positively identifying the different molecular subtypes. Table 1 shows that expression of CD95 itself, or the CD95 neighborhood signature, is most strongly associated with gene sets positively identifying MSI-type tumors. A signature reflecting immune cell infiltration^31^ – a known characteristic of MSI tumors^28^ – was also strongly correlated with CD95 expression (Table 1). CD95 expression was only weakly associated with gene signatures identifying aggressive mesenchymal/stem-like subtypes (*r*=0,10–0,19) and was inversely correlated with expression of signatures identifying epithelial-type tumors. Of the genetic parameters tested dMMR – which causes MSI – and a CpG island methylator phenotype (CIMP), were most strongly associated with CD95 expression. dMMR and CIMP are associated with MSI and the CMS1 subtype.^11, 30^ In line with these results, CD95 expression was inversely correlated with chromosomal instability (CIN), which is a hallmark of microsatellite stable CMS2-4 tumors^30^ (Table 1).

Finally, when using median CD95 and SASP expression levels as cutoff points, we found that all MSI tumors were contained within the CD95^high^-SASP^high^ quadrants using both senescence-reflecting signatures (Figures 6e and f).

## Discussion

Senescence can result from a variety of stress-inducing conditions such as telomere shortening,^32, 33^ chronic inflammation,^34, 35^ and an oxidative environment.^36^ In addition, senescence can occur in response to oncogene activation in pre-malignant cells, thereby preventing tumor formation.^29, 37, 38, 39^ However, tumors that have escaped the initial senescence response are still able to undergo senescence in response to a wide variety of cytotoxic drugs that induce oxidative stress and/or DNA damage (reviewed in refs 40, 41). In the current report, we show that, in addition to anti-cancer drugs, CD95L is also capable of inducing senescence in tumor cells by promoting chronic DNA damage. The correlation of CD95L-induced senescence with MSI/CMS1 is most likely due to the fact that such tumors express the highest levels of CD95, thereby sensitizing them to the ligand. While senescence-inducing therapy could be an effective anti-cancer treatment strategy, systemic CD95 activation is not a realistic therapeutic approach as it causes rapid and lethal liver toxicity.^42^ Interestingly, MSI/CMS1-type tumors metastasize infrequently, but the fraction that does is highly aggressive^30^ and expresses relatively high levels of CD95 (unpublished observation). MSI-type tumors are notoriously resistant to standard fluorouracil-based chemotherapy.^43^ It will therefore be interesting to explore whether the aggressive subgroup of metastatic MSI tumors are sensitive to drugs that potently induce senescence in other tumor types.^44^

Based on the pro-tumorigenic effects of CD95 in several tumor types, neutralization of CD95L is currently being tested as an anti-cancer strategy.^45, 46^ The results presented in the current report raise the question whether CD95-neutralizing therapy could interfere with senescence induction *in situ*, and if so, whether this may influence tumor growth and/or metastasis. How interference with senescence in growing or drug-treated tumors may influence tumor progression or response to therapy is currently unknown. The concept of senescent cells as pro-tumorigenic by-standers is based on studies demonstrating that co-injection of senescent fibroblasts with tumor cells accelerates tumor formation.^47, 48, 49, 50, 51^ This phenomenon is mediated by the SASP, and includes contributions of matrix remodeling enzymes, growth factors and cytokines stimulating invasion, proliferation and angiogenesis. The source-dependent heterogeneity of the SASP further suggests that its effects will be highly context dependent. Currently, very little is known about how senescent (tumor) cells modulate tumor growth *in vivo*, in particular in more advanced stages of the disease and/or after treatment. Recently developed mouse models allow the ablation of senescent cells *in vivo* and have been used to show that naturally occurring senescent cells in ageing tissue have tumor-promoting capacity as their deletion protects mice against ageing and cancer development.^52^ Such tools can now be applied to study the consequences of the generation and elimination of senescent tumor cells on tumor progression and therapy response. More specifically, these models may be applied to study how CD95L-induced senescence shapes development and progression of MSI-type tumors.

Taken together, the results identify senescence as a hitherto unrecognized consequence of CD95 signaling, particularly in MSI/CMS1-type colon tumors. Future work should address how CD95-induced senescence shapes the growth of such tumors and their response to therapy, whether the growth of other tumor (sub-) types may be regulated by CD95-induced senescence, and whether senescence-inducing therapy has value in the treatment of metastasized (aggressive) CMS1 tumors.

## Materials and methods

### Cell culture

293T, DLD1, DKO4, C26, and L13 cells were cultured in DMEM (D6429, Sigma-Aldrich, St. Louis, MO, USA) supplemented with 5% v/v FCS (F7524, Sigma-Aldrich), 2 mM ultraglutamine (BE17-605E/U1, Lonza, Walkersville, MD, USA), 50 UI/ml pen/strep (DE17-602E, Lonza) on adherent culture dishes. Patient-derived colorectal cancer colonospheres; CR16, CRC26, CRC29, CRC47, L145, L146, L167, L169, and L193^24^ were cultured in supplemented advanced DMEM/F12 (12634-010, ThermoFisher Scientific, Walthom, MA, USA) on low-adherent culture dishes. Basic-FGF (4 ng/ml) (100-18B, Peprotech, Rocky Hill, NJ, USA) was added freshly to the medium with every cell passaging. Cells were kept at 37 °C in a humidified atmosphere containing 5% CO_2_.

### Cloning and mutagenesis

CD95-ΔDD (Δ-230-314 aa) was generated from pEYFPN1-CD95 (a kind gift from Professor Dr. D Häussinger and Dr. R Reinehr) using Phusion-site-directed mutagenesis protocol (Finnzymes) according to the manufacturer's instructions and using the following pair primers phosphorylated at their 5′, forward: 5′-CTCAAGGACATTACTAGTGACTCAG-3′ and reverse: 5′-CAAGTCAACATCAGATAAATTTATTGC-3′. For cloning full-length CD95-YFP and deletion mutants into pWPT lentiviral vector: Full-length CD95-YFP, CD95-ΔDD-YFP, and CD95-ΔCyt-YFP segments were amplified from pEYFPN1-CD95, pEYFPN1-ΔDD, and pEYFPN1-ΔCyt constructs, respectively, using the following primers, forward: 5′-CGCATTTGATCACATATGGCGGCCGCGCCGCCACCATGCTGGGCATCTGGACCCTCC-3′ and reverse: 5′-CCGATTCTCGAGTTAATTAAACTAGTTTACTTGTACAGCTCGTCCATGC-3′. The PCR products were then cut with Bcl1 and Xho1, and cloned into pWPT-GFP vector (a kind gift of Professor Didier Trono) cut with BamHI and SalI to release the GFP fragment. All final constructs were sequence verified. For generation of CD95L-mcherry-C1, full-length human CD95L was amplified by PCR using the following pair of primers forward: 5′-CGCGGATCCCAGCAGCCCTTCAATTACC-3′ and reverse: 5′-CCGGAATTCGTC GACTTA GAGCTTATATAAGCCG-3′. The PCR fragment was subsequently cut with BamH1 and EcoR1 and inserted between BglII and EcoR1 sites in mcherry-C1 (Clontech, Mountain View, CA, USA). hCD95 shRNA (TRCN0000038696) insensitive versions of full-length CD95-YFP and mutant ΔDD-YFP were constructed using Phusion-site-directed mutagenesis protocol. The TRCN0000038696 target sequence GTGCAGATGTAAACCAAACTT was mutated to GTGCAGGTGTAAGCCAAACTT without amino-acid sequence change using the following pair of primers phosphorylated at their 5′: forward: 5′-CCAG AAT ACC AAG TGC AGG TGT AAG CCA AAC TTT TTT TGTAAC-3′ and reverse: 5′-G TCC GGG TGC AGT TTA TTT CCA CTT CTA AGCCAT GTCC-3′.

### Plasmids and lentiviral transduction

For lentiviral production, 293T cells were transfected using calcium phosphate method with third-generation packaging system (gift from Professor D Trono). For generation of stable knockdown lines, cells were infected with control shRNA (pLKO.1-puro-sh-Scramble) or two validated shRNA-targeting human CD95: pLKO.1-puro-shCD95 #92 (TRCN0000038694) and pLKO.1-puro-shCD95 #94 (TRCN0000038696). For rescue experiments, CD95 knockdown cells were transduced with pWPT-YFP-CD95-FL expressing full-length CD95 or a mutant lacking the death domain (pWPT-YFP-CD95 ΔDD). For generation of stable colonospheres lines expressing control mcherry or mcherry-CD95L, CRC29 cells were transduced with pWPT-mCherry or pWPT-mCherry-CD95L lentiviral vectors. To create a p53 knockdown lines, cells were transduced with pLKO.1-GFPshp53 as well as pLKO.1-puro-shp53 (respectively, pLKO.1-GFP and pLKO.1-puro-shSCR were used to make control lines). Cells transduced with plasmids containing a puromycin-resistance gene where selected by addition of puromycin to the culturing medium.

### Antibodies and reagents

p53 (DO-1, #sc-126, Santa Cruz, Dallas, TX, USA), p21Waf1 (DF10, #OP68, Merck Millipore, Darmstadt, Germany) was used for western blotting, p21 (C-19, #sc-397, Santa Cruz) was used for immunofluorescence, cleaved caspase-3 (Asp175, #9661, Cell Signaling, Danvers, MA, USA), p-histone H2A.X (Ser139, JBW301, #05-636, Merck Millipore), iCAD/DFF (Cleaved form, #ABC27, Merck Millipore), CD95/FAS (B-10, sc-8009, Santa Cruz), anti-CD95 clone CH11 (#05-201, Merck Millipore), *β*-catenin (610154, BD, Franklin Lakes, NJ, USA), β-Actin (AC-15, #NB600-501, Novus Biologicals, Littleton, CO, USA), GaM IgG-HRP (#1706516, Bio-Rad, Hercules, CA USA), GaR IgG-HRP (#170-6515, Bio-Rad, Hercules, CA, USA), GaR IgG-Alexa Fluor 488 (#A11034), GaR IgG-Alexa Fluor 568 (#A11036), GaM IgG-Alexa Fluor 568 (#A11031), and GaM IgG-Alexa Fluor 647 (#A21236) were all purchased from ThermoFisher Scientific. Z-VAD-FMK (FMK001, R&D Systems, Minneapolis, MN, USA). Propidium iodide (PI) (p4170), RNase A (R5000), and 5-carboxyfluorescein diacetate succinimidyl ester (CFSE, 21888) were all purchased from Sigma-Aldrich.

### FC-CD95L production

293T cells were transfected with pCDNA3.1-FC-CD95L. The following day, the cells were washed with PBS and new medium containing 0.2% FCS was added. After 2 days, the supernatant was collected and filtered through a 0.45 and 0.2 *μ*m filter consecutively. The FC-CD95L containing medium was incubated o/n with Protein A Agarose (11134515001, Roche, Basel, Switzerland) at 4 °C. The FC-CD95L was released from the beads by resuspending them in 100 mM glycine pH 2.5. The supernatant was collected and neutralized with 1 M Tris pH 9.0. To concentrate the ligand, the solution was pipetted onto an Amicon Ultra-4 Centrifugal Filter Unit with 30 kDa pore-size membrane (UFC803024, Merck Millipore) and spun down according to the manufactures' protocol. Quality control and bioactivity of the FC-CD95L was assessed by coomassie staining and apoptotic capacity in DKO4 cells using MTT assay (475989, Merck Millipore). Membrane-bound CD95L (FasL, #01-210, Merck Millipore) was used (10ng/ml) as a positive control and reference.

### CD95L treatment regimen

The colonospheres were dissociated with trypsin (T4174, Sigma-Aldrich), and cultured in the presence of FC-CD95L or FC control (401104, Merck Millipore). The culture medium was refreshed 2–3 × per week. The cells were pretreated for 2 weeks (cycle I), followed by another 2-week treatment cycle (cycle II).

### Colony-forming assay

Following 2 weeks of pre-treatment with FC-CD95L or FC control, the colonospheres were dissociated and plated at 100 cells/20 *μ*l Matrigel (354234, BD) 50% v/v solution per 48-well. After polarization of the Matrigel at 37 °C, the wells were filled with medium supplemented with 0.2 mM and 10 ng/ml FC-CD95L or FC control in the presence or absence of *N*-acetylcysteine. The medium was refreshed 2 × a week. The number of colonies were counted after 2 weeks of culturing using an inverted microscope (Nikon Eclipse TS100).

### SA-*β*GAL

Colonospheres were dissociated and treated with FC-CD95L or FC control at days 0, 2, and 5. Standard SA-*β*GAL assay was performed at day 7. For 3D cell culture, colonospheres were fixed in 4% formaldehyde (4078-9020, Klinipath, Duiven, the Netherlands) for 20 min at room temperature while rotating. Colonospheres were washed 2 × with wash buffer (40 *μ*m citric acid/sodium phosphate pH 6 with 150 *μ*m NaCl), resuspended in assay buffer and incubated in the dark for 6–20 h in a 37 °C incubator w/o CO_2_ heat block. Colonospheres were washed with demi-water and centrifuged on a glass slide using cytospin 4 (ThermoFisher Scientific). The slides were air-dried and mounted with a coverslip and Prolong Gold Antifade Mountant (P36930, ThermoFisher Scientific). At least 10 pictures were taken per condition (EVOS XL, ThermoFisher Scientific) and analyzed by counting the number of white *versus* blue cells. L13 cells were treated with FC-CD95L or FC control at days 0, 2, and 5. On day 5, cells were plated on 12 mm coverslips in a 24-well plate and fixed in 4% formaldehyde on day 7 (10 min at 37 °C). The cells were processed for *β*-galalctosidase staining as described above.

### Western blot analysis

FC control and FC-CD95L-treated colonospheres were collected at the indicated time points and lyzed in laemmli lysis buffer (2.5% SDS, 20% glycerol, 120 mM Tris pH 6.8). Equal amounts of protein were run on SDS-PAA gels and were analyzed by western blot (Trans-Blot Turbo, Bio-Rad, Hercules, CA, USA).

### Immunofluorescence

FC control and FC-CD95L-treated colonospheres were fixed in 4% formaldehyde (20 min at room temperature with rotation), permeabilized in 100% ice-cold methanol and stored at −20 °C for a minimum of 16 h. The samples were blocked in PBS/5% BSA/0.1% Tween-20 (30 min at room temperature with rotation) and the primary antibodies were added (in PBS/2% BSA/0.1% Tween-20 for 2 h at room temperature with rotation). Finally, the samples were incubated with the secondary antibodies and Dapi (D9542, Sigma-Aldrich) (1 h at room temperature with rotation in the dark). The cells were placed on a glass slide in a droplet of ethanol, air-dried and mounted with ProLong Gold Antifade Mountant and a coverslip. The slides were analyzed with confocal microscope (Zeiss LSM510).

### CD95L neutralization experiment

A CD95L neutralizing antibody was made via the recombinant baculovirus/insect cell system with the expression vector pCS1392-mFas-FcIgG1 (kindly provided by François Godeau, ref. 3). It comprises of an fc-tagged extracellular ligand-binding domain of the mouse CD95 receptor referred to as mCD95. The efficiency to block CD95L-induced apoptosis in DKO4 cells was tested with a MTT assay. Prior to adding the FC-CD95L conditioned medium (=supernatant of colonosheres grown and treated in Matrigel) to the cells or colonospheres, it was pre-incubated for 10 min with 1 *μ*g/ml mCD95 or control to neutralize all present ligand.

### Cytokine array

For the cytokine array, supernatants of FC-CD95L or FC control-treated CRC29 in Matrigel were pooled and concentrated (spun down on an Amicon Ultra-4 Centrifugal Filter Unit with 3 kDa pore-size membrane (UFC800324, Merck Millipore), according to the manufacturer's instructions). Next, a cytokine array (Biotin label-based human antibody array I, #AAH-BLM-1-4, RayBiotech, Norcross, GA, USA) was performed according to the manufacturer's instructions.

### Luminex analysis

CRC29-shSCR, CRC29-shp53, L169, and L193 were pretreated with 10 ng/ml FC-CD95L for 7 days. After pre-treatment, cells were plated in Matrigel and conditioned medium was collected at the indicated time points, and analyzed at the multiplex core facility by Luminex (multiplexed FACS). DLD1 and DKO4 cells were treated with control FC, or with increasing concentrations of FC-CD95L (2, 5, and 10 ng/ml). Conditioned medium was collected at the indicated time points, and analyzed by Luminex.

All assays were thoroughly validated. http://www.umcutrecht.nl/en/Hospital/Professionals/Diagnostiek-aanvragen/Laboratory-for-Translational-Immunology/Multiplex-Facility).

### Software

Optical density, fluorescent intensity, and cell count measurements were obtained with ImageJ Software 1.50c (Wayne Rasband, National Institutes of Health, USA). Statistical analysis was done with Graphpad Prism 6 (Dr. Harvey Motulsky, San Diego, CA, USA).

### Cell cycle analysis

Cells were dissociated with trypsin. Single cells were fixed in ice-cold 70% ethanol for at least 15 min on ice. DNA content was stained with 20 *μ*g/ml PI in PBS buffer supplemented with 100 *μ*g/ml RNase A for 30 min at 37 °C, and analyzed by flow cytometry (BD FACSCalibur, Breda, The Netherlands).

### CFSE staining

CFSE was used to stain cells and monitor the number of cell divisions. Single cells were resuspended in PBS containing 0.1% FCS w/o Ca^2+^ or Mg^2+^ at cell density of 10 × 10^6^ cells/ml. CFSE was added at final concentration of 1 mM and incubated for 10 min at 37 °C, with agitation every 2 min. The reaction was blocked with an excess of cold medium containing 20% FCS and incubated on ice for 5 min. After washing, cells were resuspended in their own culture medium and equally divided over the experimental conditions. At the indicated time point, cells were dissociated and resuspended in PBS containing 5 *μ*g/ml PI and measured immediately on a flow cytometer (BD FACSCalibur). The dead cells (PI positive) were excluded from the analysis using cell Quest software (BD).

### CD95 staining

Colonospheres were dissociated and blocked in PBS/5% FCS/0.2% sodium azide (30 min at 4 °C with agitation). The samples were stained with CH11 anti-CD95 antibody in PBS/1% FCS/0.2% sodium azide (30 min at 4 °C with agitation). After washing, cells were incubated with GaM IgG-Alexa Fluor 647 secondary antibody for 30 min at 4 °C with agitation in the dark. To exclude dead cells, PI (p4170, Sigma-Aldrich) was added prior to the measurement on a flow cytometer (BD FACSCalibur).

## Figures and Tables

**Table 1:**
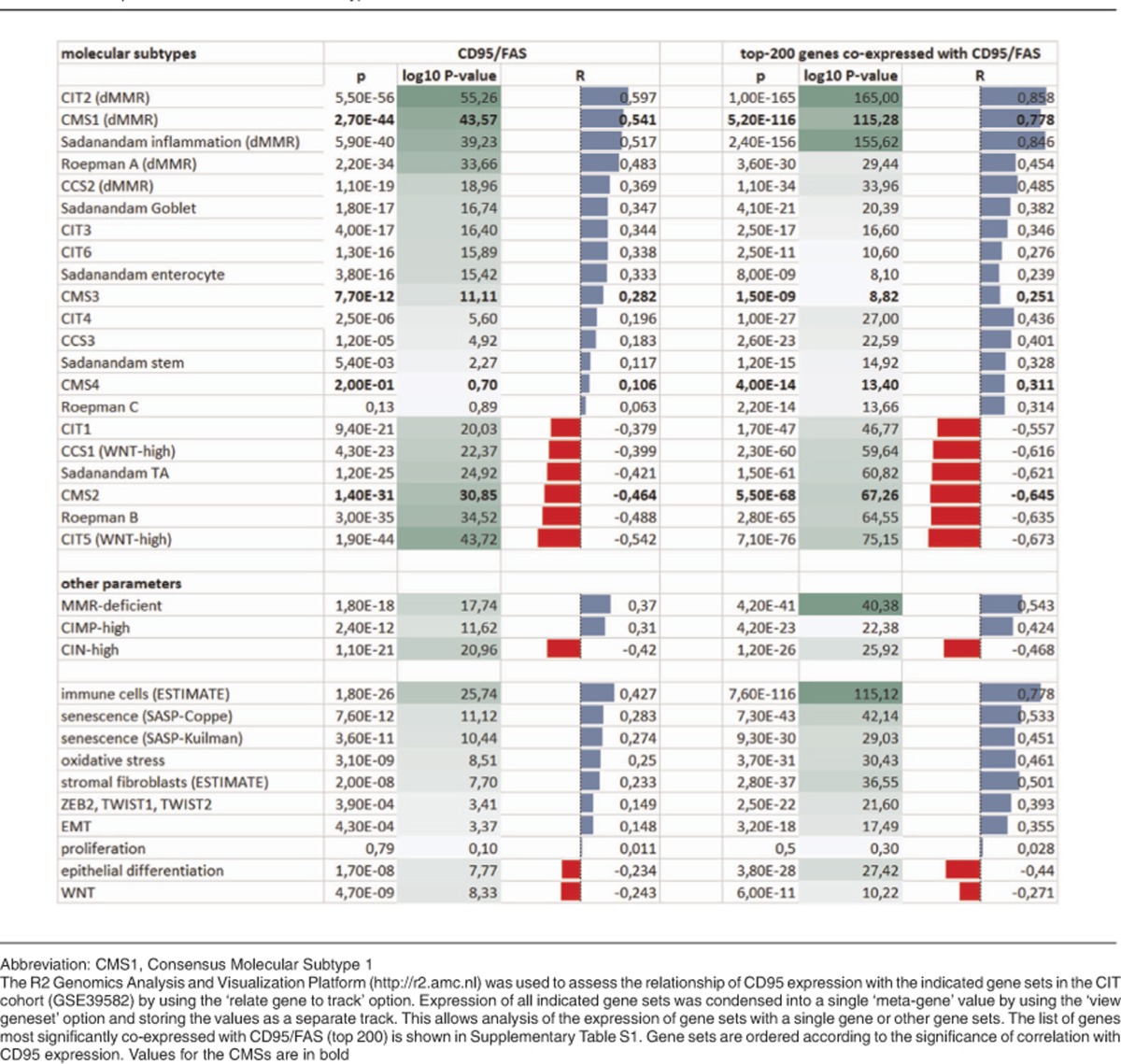
CD95 expression is associated with MSI-type CRC
