# Supplementary material for: CD95 ligand induces senescence in mismatch repair-deficient human colon cancer via chronic caspase-mediated induction of DNA damage
Source: Cell Death Dis. 2017 Mar 16;8(3):e2669–. doi: 10.1038/cddis.2017.87 (PMC5386578; doi:10.1038/cddis.2017.87)
Supplement: Supplementary Figures [file cddis201787x1.pdf]

Figure S1

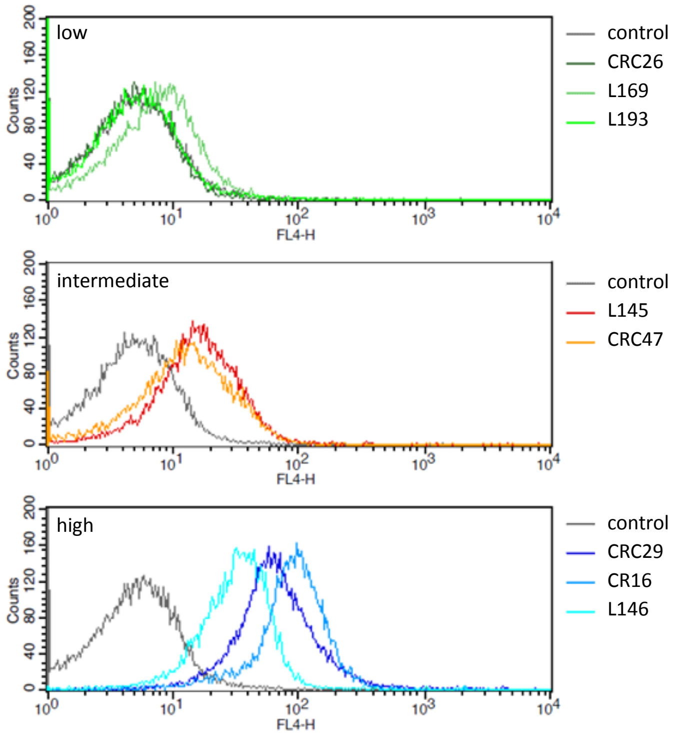

Figure S1. Examples of FACS plots of CH11 and control stainings for cell surface CD95 expression on 11 colonosphere lines.

Figure S2

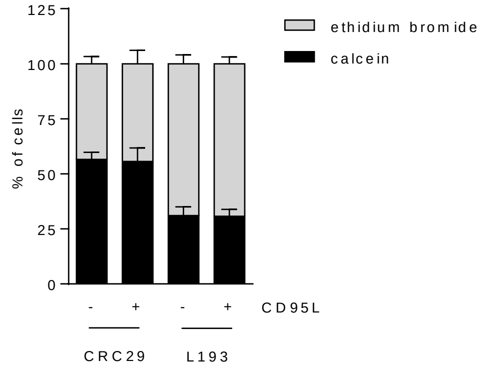

Figure S2. Colonospheres were treated with FC or FC-CD95L for 14 days, plated in matrigel with or without FC-CD95L. The cells were stained with calcein and ethidium bromide on day 3 after plating in matrigel. The number of green versus red cells were counted using an EVOS FL cell imaging system.

Figure S3

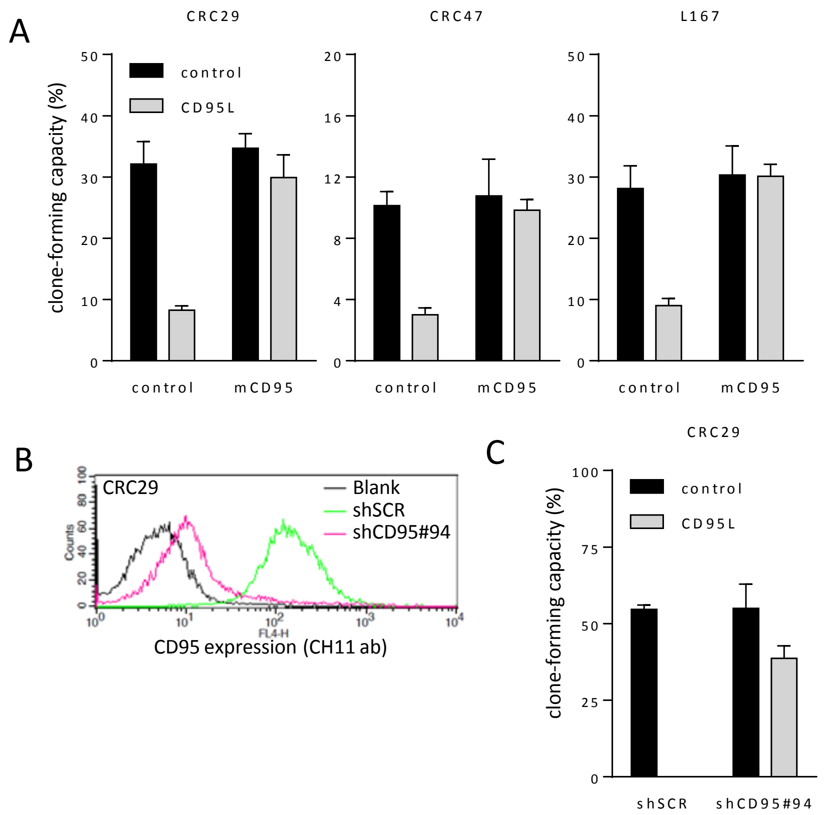

Figure S3. (A) The extracellular part of murine CD95 (mCD95) was used to trap CD95L and prevent its signaling. Colonospheres were plated for colony-forming capacity as described in figure 1A. mCD95 completely rescued CD95L reduced clonogenicity. **(B)** FACS analysis of CD95 receptor expression in colonospheres stably expressing shRNAs targeting CD95 mRNA or a srambled version thereof, using the CH11 antibody. **(C)** CD95-suppression rescues CD95L induced loss of clonogenic potential.

Figure S4.

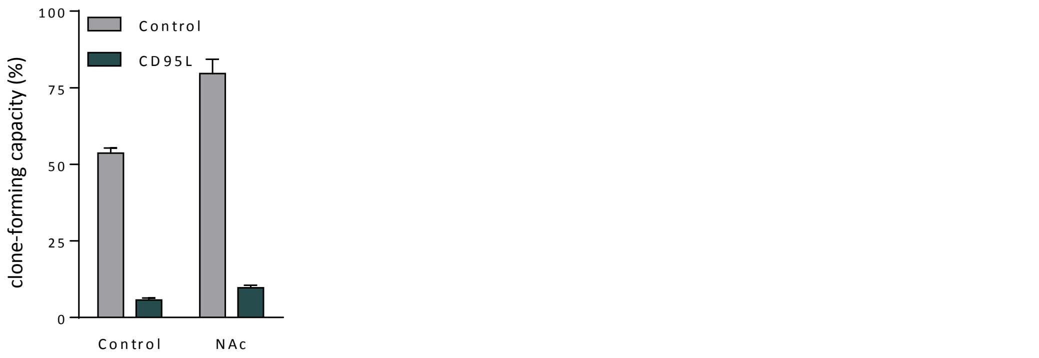

Figure S4. CRC29 colonospheres were made single cells, plated in matrigel, and treated with either FC-control or FC-CD95L in the presence or absence of 2,5mM N-acetyl cysteine (NAc).

Figure S5.

A

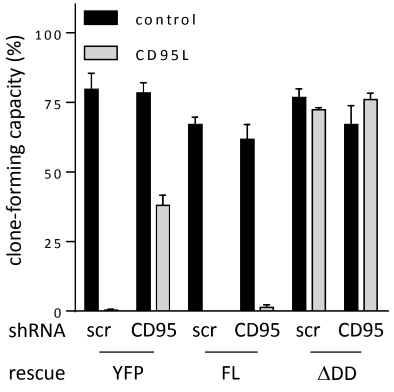

B

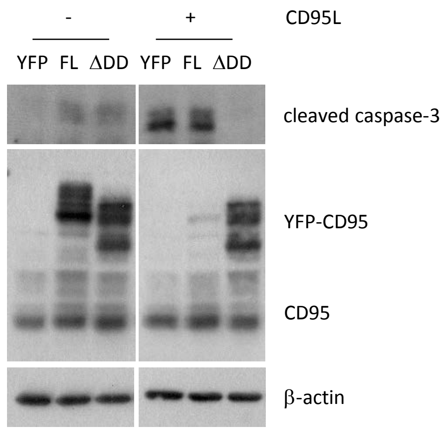

Figure S5. **(A)** CRC29 colonospheres stably expressing shRNAs targeting human CD95 or a srambled version thereof were rescued by expression of shCD95-insensitive mutants of either full-length or death-domain-deleted CD95 fused to YFP, or YFP alone. Cells were then exposed to FC-CD95L and clonogenic capacity was tested as in Figure 1A. **(B)** CRC29 colonospheres treated as (a) were analyzed for caspase-3 processing using Western blot analysis.

Figure S6.

A

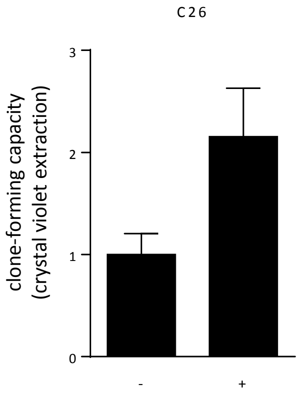

B

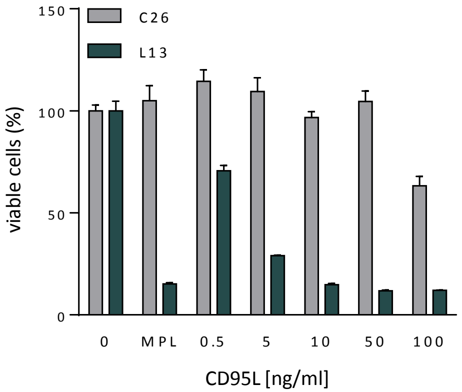

C

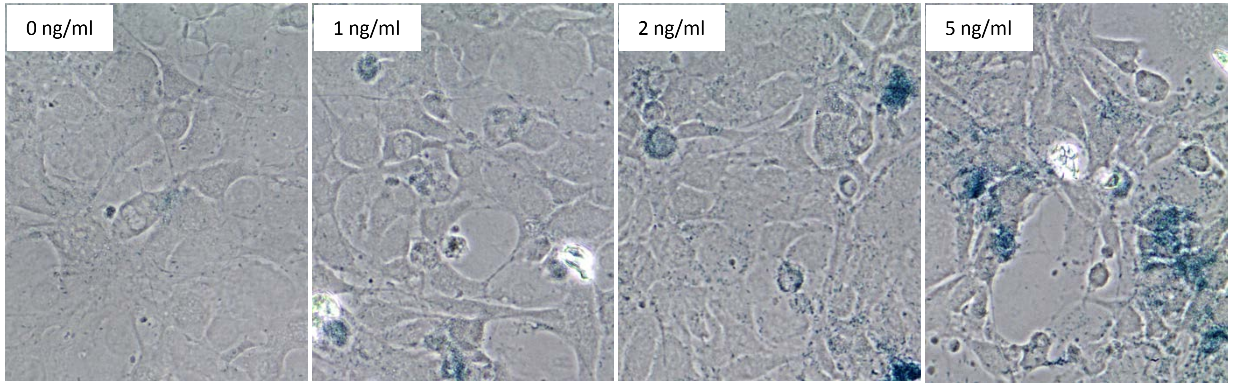

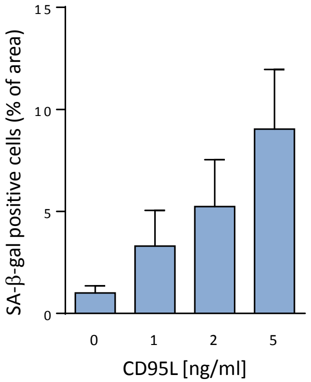

Figure S6 **(A)** Murine C26 colorectal cancer cells were treated with 10ng/ml CD95L for 2 weeks, after which they were plated in matrigel and treated for another week. The cells were then fixed and stained with Crystal Violet. The color of the dye was extracted with acetic acid and absorbance at 560nm was measured. **(B)** MTT assay of KRAS mutant (C26) and KRAS wt (L13) colorectal cancer cells treated with the indicated concentrations of FC-CD95L and 10ng/ml commercial CD95L (Millipore, membrane-bound (MPL)). Cell viability is depicted as % of control cells after 48h of treatment. **(C)** L13 cells were treated for 7 days with the indicated FC-CD95L concentrations, after which they were analyzed for senescence-associated β-GAL activity (upper images). The percentage of area of senescent cells was calculated from 10 different photos per condition using ImageJ software (lower graph) .

Supplemental Table legends

Supplemental Table 1. Statistics for the results in Figures 4 and 5.

Supplemental Table 2. Genetic characteristics of the spheroid lines used.

Supplemental Table 3. The CD95L-induced inflammatory response (CIR) gene list.

Supplemental Table 4. The top 200 FAS/CD95-co-expressed genes. All genes that were positively associated with expression of CD95/FAS in both the CIT566 and MVRM cohorts were identified using a single-gene cutoff value of p<E-6. Gene Venn was then used to identify the 200 most significantly CD95-co-expressed genes in both cohorts.
